# Response to growth hormone therapy and gonadal pathology in 45,X/46,XY females

**DOI:** 10.1186/1687-9856-2015-S1-O54

**Published:** 2015-04-28

**Authors:** Angela Titmuss, Paul Benitez-Aguirre, Andrew Biggin, Maria Craig, Bin Moore, Neville Howard, Christopher Cowell, Geoffrey Ambler, Shubha Srinivasan

**Affiliations:** 1 Institute of Endocrinology and Diabetes, The Children’s Hospital Westmead, Sydney, NSW, Australia; 2 Discipline of Paediatrics and Child Health, University of Sydney, Sydney, NSW, Australia

## 

Turner syndrome (TS) and related sex chromosome abnormalities are associated with a variety of karyotypes and phenotypes affecting 1 in 2500 live births. Mosaicism with Y material (45,X/46,XY) and female phenotype is rare (<1 in 15 000 births)[1]. Their risk of gonadal malignancy is 10-15%, and up to 50% in those with ambiguous phenotype at birth[2]. The SHOX gene is located on both X and Y chromosomes but is more prone to deletions on the X chromosome, potentially influencing height outcomes across TS karyotypes[3]. However, children with SHOX deficiency respond similarly to TS girls when treated with the same dose of growth hormone (GH)[4]. We therefore examined height outcomes and gonadal malignancy rates in TS vs 45,X/46,XY females.

We identified 198 females aged ≤ 30 years with TS or mixed gonadal dysgenesis treated with GH (under TS or auxological criteria). Final height (FH) was available on 51 TS (45,X or mosaic without Y material) females. An additional 13 had 45,X/46,XY karyotype with TS phenotype, and two had non-mosaic 46,XY karyotypes with cytogenetic abnormalities consistent with TS. Of these 15 females, gonadal tissue histology was available for 11 and FH in nine. We evaluated patient records for age, height, mid-parental height (MPH), GH dose at commencement, duration of therapy and growth response at 12 months and at FH. Comparisons between TS and 45,X/46,XY groups were performed using the Mann-Whitney U test.

All 45,X/46,XY patients had a female phenotype and five had clitoromegaly at birth. Three were identified prenatally; age at diagnosis ranged from birth to 13 years, with the most common presenting features being short stature (n=5), ambiguous genitalia (n=5) and dysmorphic features (n=2). Of the 11 that underwent gonadectomy, four (none virilised at birth) had a gonadoblastoma, including one dysgerminoma in situ.

Age, height, MPH,GH dose at commencement, duration of therapy and height z-score after 12 months did not differ between groups. Median FH z-score for 45,X/46,XY was higher than TS, -1.12 [range -1.96,0.31], vs -1.59 [-3.12,0.01], p=0.016. Response to GH therapy (median Δ height z-score) after 12 months was similar: 0.45 [-0.04,0.84] vs 0.39 [-0.21,1.14], p=0.81. However, height response over the total duration of therapy was better for 45,X/46XY: 1.5 [0.72,2.88] vs 0.87 [-0.98,2.14], p=0.009.

45,X/46,XY females appear to respond differently to GH therapy, suggesting a possible contribution of SHOX on the Y chromosome. The rate of germ cell tumours in non-virilised females (36%) is higher than previously reported.

## References

[B1] Lindhart Johansen M Hagen CP Rajpert-De Meyts E KjÃ¦rgaard S Petersen BL SkakkebÃ¦k NE 45,X/46,XY Mosaicism: Phenotypic Characteristics, Growth, and Reproductive Function – a Retrospective Longitudinal Study J Clin Endocrin Metab 2012 97 8 E1540 E1549 10.1210/jc.2012-1388 22605431

[B2] Cools M Pleskacova J Stoop H Hoebeke P Van Laecke E Drop SL Gonadal Pathology and Tumor Risk in Relation to Clinical Characteristics in Patients with 45,X/46,XY Mosaicism J Clin Endocrin Metab 2011 96 7 E1171 E1180 10.1210/jc.2011-0232 21508138

[B3] Oliveira CS Alves C The role of the SHOX gene in the pathophysiology of Turner syndrome Endocrinol Nutr 2011 58 8 433 442 10.1016/j.endonu.2011.06.005 21925981

[B4] Blum WF Ross JL Zimmermann AG Quigley CA Child CJ Kalifa G GH Treatment to Final Height Produces Similar Height Gains in Patients with SHOX Deficiency and Turner Syndrome: Results of a Multicenter Trial J Clin Endocrin Metab 2013 98 8 E1383 E1392 10.1210/jc.2013-1222 23720786

